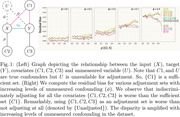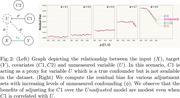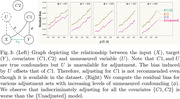# Indiscriminate adjustment for confounders is worse than you think and what can be done about it

**DOI:** 10.1002/alz.090913

**Published:** 2025-01-09

**Authors:** Harsh Sinha, Pradeep Reddy Raamana

**Affiliations:** ^1^ University of Pittsburgh, Pittsburgh, PA USA

## Abstract

**Background:**

Training a “robust” predictive model is a non‐trivial task, especially for observational datasets. Datasets often contain confounding variables which must be de‐confounded (also less accurately referred to as “regressed out”) to eliminate the bias in predictive models. Due to the inherent uncertainty and complexity that surrounds the identification of true confounders, typically, all such covariates are regressed out indiscriminately. The prevailing wisdom is that adding more covariates cannot harm the predictive model, rather omitting measured covariates can lead to bias. But, whether inclusion/exclusion covariate has good/bad influence is contingent on the dataset. Therefore, confounding bias induced by an adjustment set must be empirically quantified rather than selecting covariates based on heuristic criteria. We demonstrate an approach to quantify confounding bias that is universally applicable to various machine learning (ML) algorithms facilitating evidence‐based decision making for covariate selection.

**Method:**

In this work, we focus on restricted permutation tests to quantify the confounding bias in ML models. Specifically, the bias is evaluated by summing up the conditional association of residualized features (X) and the target (Y) for an adjustment set C (referred to as residual bias) under the null hypothesis. Using simulated data, we analyze the post‐adjustment residual bias for Random Forest model using various adjustment sets across multiple scenarios.

**Result:**

We demonstrate that 1) adjustment for inappropriate covariates can increase existing confounding bias 2) adjusting for a covariate acting as a mere proxy for a confounder may not be beneficial and 3) finally, adjusting for a true confounder can even increase bias.

**Conclusion:**

We don’t advocate the use of independent criteria to select covariates individually. It is possible that bias induced by one covariate is suppressed or amplified by another covariate. Therefore, we recommend that confounding bias for each adjustment set should be quantified rather than choosing covariates based on availability or out‐of‐convenience. Such choices may enhance predictive accuracy in the training sample but can lead to biased predictions in the population‐of‐interest.